# Expression Patterns of Microenvironmental Factors and Tenascin-C at the Invasive Front of Stage II and III Colorectal Cancer: Novel Tumor Prognostic Markers

**DOI:** 10.3389/fonc.2021.690816

**Published:** 2021-08-19

**Authors:** Mai Hashimoto, Noriyuki Uesugi, Mitsumasa Osakabe, Naoki Yanagawa, Koki Otsuka, Yoshiki Kajiwara, Hideki Ueno, Akira Sasaki, Tamotsu Sugai

**Affiliations:** ^1^Department of Molecular Diagnostic Pathology, School of Medicine, Iwate Medical University, Shiwagun’yahabachou, Japan; ^2^Department of Surgery, School of Medicine, Iwate Medical University, Shiwagun’yahabachou, Japan; ^3^Department of Surgery, National Defense Medical College, Tokorozawa, Japan

**Keywords:** cancer-associated fibroblast, colorectal cancer, cluster analysis, prognostic marker, tenascin-C

## Abstract

**Background:**

Biological markers expressed in cancer cells and the surrounding cancer-associated fibroblasts (CAF) can be used for prediction of patient prognosis in colorectal cancer (CRC). Here, we used immunohistochemical techniques to evaluate cancer cells’ expression of specific biomarkers that are closely associated with neoplastic progression.

**Methods:**

Immunohistochemical markers included Ki-67, p53, β-catenin, MMP7, E-cadherin and HIF1-α. We also characterized microenvironmental markers expressed by CAF, including expression of α-smooth muscle actin, CD10, podoplanin, fibroblast specific protein 1, platelet derived growth factor β, fibroblast association protein, tenascin-C (TNC), ZEB1 and TWIST1. The study population consisted of 286 CRC patients with stage II and III disease. Stage II and III CRC were divided into a first and a second cohort (for validation). The CRCs were stratified using cluster analysis. To identify the utility of prognostic markers in stage II and III CRC, univariate and multivariate analyses were performed in both cohorts.

**Results:**

Stage II and III CRCs were stratified into 3 subgroups. Specific subgroups were significantly correlated to disease-free survival using univariate and multivariate analyses in the first cohort. High expression of TNC was identified as a single prognostic marker in both cohorts by univariate and multivariate analyses.

**Conclusions:**

We suggest that the presence of a specific subgroup defined by multiple markers can be used for prediction of CRC outcome in stages II and III. In addition, we showed that high expression of TNC was correlated with a poorer prognosis in stages II and III of CRC.

## Introduction

Colorectal cancer (CRC) is the third most commonly diagnosed cancer and the third leading cause of cancer death in both men and women in the United States (1). These trends for incidence and mortality are common worldwide (1). Remarkable progress has been made in the diagnosis and treatment of CRC. In spite of such advances, CRC is often discovered at an advanced stage at which point achieving a cure is very difficult (2). Therefore, the development of effective markers to predict patient prognosis of CRC is greatly needed.

The outcome of patients with CRC can be predicted by prognostic factors, such as the TNM staging system proposed by the UICC and AJCC (3, 4). Additionally, novel and promising prognostic biomarkers are listed in the WHO classification 2019 (5). There are 2 histological processes that are present within the tumor microenvironment at the invasive front of CRC: tumor budding and the desmoplastic reaction (DR) (6–9). Tumor budding is defined as single cells or clusters of up to four tumor cells at the invasion front of CRC (6–8). It is closely associated with both local and distant metastases and is therefore a histological biomarker of tumor progression and a poor prognosis (6–8). The classification of the DR was recently proposed by Ueno et al. as a prognostic histological marker (9). A pronounced desmoplastic stromal reaction in the microenvironment involves complex cellular interactions at the invasive front (10). This theory posits that cooperation between cancer cells and cancer associated fibroblasts (CAFs) present within the tumor microenvironment is necessary to support tumor growth and progression (10, 11). In addition, the microenvironment itself plays an important role in neoplastic progression and metastasis in CRC (10, 11). Whereas such histological findings are widely used as markers for establishing a patient’s prognosis, they do not explain the underlying cellular processes that promote tumor growth and metastasis (12, 13). Therefore, the discovery of additional markers would be very beneficial. We propose that identification of protein expression patterns in cancer cells and CAFs could provide new biological insights and guide the development of new therapies for CRC (12, 13).

In this study, we analyzed immunohistochemical data to identify possible protein expression patterns in stages II and III of CRC that predict patient outcome. We focused on markers that are closely associated with tumor growth and progression within the microenvironment.

## Materials And Methods

### Patients

CRC patients who underwent curative surgery at stages II or III at Iwate Medical University Hospital from January 2009 to December 2015 were included in the present study. In total, 286 patients were included the first cohort (148 cases) and in a second cohort for validation (138 cases), which were evaluated through a retrospective analysis. We used a block randomization method in the research design to select and divide participants into different groups or conditions in order to avoid bias in the selection of two cohorts. Paraffin embedded tissues were well preserved, medical records were complete and patient status had been followed up, including overall survival and disease-free survival data that were confirmed through telephone interviews and by the mail. In addition, cases with invasion beyond the proper muscular layer were included for determination of the desmoplastic reaction (9). Finally, patients who underwent preoperative chemoradiotherapy and emergency surgery were excluded. In addition, patients who had evidence of hereditary non-polyposis colorectal cancer or familial adenomatous polyposis were not enrolled. The clinicopathological variables characterizing the patients included tumor location, stage and t stage, histological type, lymphatic/venous invasion and tumor budding. The variables were recorded according to the General Rules for Management of the Japanese Colorectal Cancer Association (**Table 1**) (14). In addition, DR classification was determined based on Ueno’s classification (9).

**Table 1:** Clinicopathological findings in stage II and III colorectal cancer.

		Cohort 1 (%)		Cohort 2 (%)		
Total		148		138		*p* value
Age, median (range) (y)		67.5 (34–94)		70.0 (41–88)		0.1583
Sex	Man	90 (60.8)		81 (58.7)		0.7193
	Woman	58 (39.2)		57 (41.3)		
Location	Right colon	30 (20.3)		32 (23.2)		0.4271
	Left colon	64 (43.2)		49 (35.5)		
	Rectum	54 (36.5)		57 (41.3)		
pT	pT3	129 (86.5)		111 (80.4)		0.1474
	pT4	19 (13.5)		27 (19.6)		
Stage	II	71 (48.0)		69 (50.0)		0.8129
	III	77 (52.0)		69 (50.0)		
Histological type	WDA	17 (11.5)		26 (18.8)		0.0681
	MDA	121 (81.8)		109 (79.9)		
	PDA	2 (1.4)		1 (0.7)		
	PAP	6 (4.1)		1 (0.7)		
	MUC	2 (1.4)		1 (0.7)		
Lymphatic invasion	Positive	130 (87.8)		129 (93.5)		0.4404
	Negative	18 (12.2)		9 (6.5)		
Venous invasion	Positive	129 (87.2)		128 (92.8)		0.4534
	Negative	19 (12.8)		10 (7.2)		
Tumor budding	Low	117 (79.1)		108 (78.3)		0.8861
	High	31 (20.9)		30 (21.7)		
Desmoplastic reaction	Mature	65 (43.9)		61 (44.2)		0.9877
	Intermediate	53 (35.8)		49 (35.5)		
	Immature	30 (20.3)		28 (20.3)		
Disease-free survival, median (range) (d)		1857 (33–3196)		1835 (93–3308)		
Overall survival, median (range) (d)		3077 (52–3196)		2195 (93–3308)		

WDA, well-differentiated adenocarcinoma; MDA, moderately differentiated adenocarcinoma; PDA, poorly differenced adenocarcinoma; PAP, papillary carcinoma; MUC, mucinous carcinoma.

This study was approved by the local ethics committee of Iwate Medical University (approval number MH2020-070), and all patients provided informed consent.

#### Determination of Disease-Free Survival

We determined the duration of disease-free survival at which metastasis was discovered during the follow-up period (2 times/year to 3 times/year) using computed tomography.

### Chemotherapeutic Treatment After Surgery for Stage II or III CRC

Following surgery, Capecitabine or UFT/UZEL (Tegafur Uracil + Calcium Folinate) were administered in stage II CRC (20/140 cases), whereas FOLFOX, including the drugs leucovorin calcium (folinic acid), fluorouracil and oxaliplatin were used in stage III CRC (85/146 cases). The other 181 patients, including 120 cases in stage II and 61 cases in stage III did not receive additional chemotherapy following surgery.

#### Determination of Sample Size

The sample size required to identify differences in overall and disease-free survival between cohorts was determined using JMP Pro 13.0 software (SAS, Tokyo, Japan). From the calculation, at least 120 cases were required. The statistical power (detection power) was set to 0.8, which is commonly used in medical studies.

### Tissue Microarray Construction (TMA)

The TMAs were assembled using a manual tissue array (Azumaya Co, Tokyo, Japan). Five mm tissue cores were taken from each targeted lesion and placed into a recipient block containing 12 cores including 10 cancer tissues and 2 cores for control tissues (normal colon; CRC). After construction, 3-micron sections were cut and stained with hematoxylin and eosin on the initial slides to verify the histologic diagnosis. Serial sections were cut from the TMA block for immunohistochemical staining.

### Immunohistochemistry

Tumors were routinely fixed in 20% neutral-buffered formalin and embedded in paraffin wax. Three-micron-thick paraffin sections were cut, dewaxed, and rehydrated. Microarray slides were incubated in 3% hydrogen peroxide to block endogenous peroxidase. Antigen retrieval was performed using an autoclave-based method, followed by incubation with the primary antibody overnight at 4°C in a high humidity cabinet. Slides were processed using the Dako Autostainer Universal Staining System (Dako, Glostrup, Denmark) (12). The specimens were treated with citrate buffer (pH 6.0) using a microwave [three times for 5 min, 750 W; cat. no. H2500; Microwave Processor (Bio-Rad Laboratories, CA, USA)] and then reacted with antibodies, as previously described. Antibodies used in this study were classified into 2 subgroups: epithelial (cancer cells) and interstitial (cancer associated fibroblasts, CAF) markers. Antibodies targeting CAFs included the following: α-smooth muscle actin (α-SMA, Dako 1A4), CD10 (Dako, 56C6), podoplanin (Dako, D2-40), fibroblast specific protein 1 (FSP1; S100A4, Dako, polyclonal), platelet derived growth factor receptor (PDGFR-β; 28E1, Cell Signaling Technology), fibroblast association protein (FAP, Abcam, EPR20021) and tenascin-C **(**IBL, 4F10TT**)**. For EMT, we utilized zinc finger E-box binding homeobox 1 (ZEB1, Sigma-Aldrich, polyclonal) and Twist-related protein 1 (TWIST1, Abcam, Twist2C1a). CAFs were recognized as “spindle-shaped cells” by experienced pathologists (T.S. and N.U.). Cytoplasmic staining of tumor cells was conducted with antibodies against α-SMA, CD10, podoplanin, FSP1, PDGFR-β, FAP and tenascin-C. Nuclear staining of fibroblasts was based on positivity for ZEB1 and TWIST1 expression. Furthermore, antibodies targeting cancer cells in this study included Ki-67 (Dako, MIB1) for proliferative activity, p53 (Dako, Do7) for p53 mutation, β-catenin (Dako, β-catenin-1) for activation of Wnt signaling, a central signal transducer in CRC, MMP7 (Daiichi Fine Chemical, 141-7B2) for cancer progression, E-cadherin (Dako, NCH-38) for cellular adhesion and HIF1-α (Novus Biologicals, polyclonal) for cancer-specific metabolic marker which may be associated with tumor progression. Detailed information of antibodies is summarized in **Supplementary Table 1**.

### Assessment of Scoring of Immunohistochemical Expression

The expression of the markers was scored for both the intensity and extent of immunopositivity, as described in a previous report with slight modification (15). The immunostaining intensity of the cancer cells and CAFs in the CRCs was classified into 4 categories as follows: negative, weak, moderate and strong. The immunostaining extent was semi-quantified as follows: 0%, 1-25%, 26-50%, 51-100%. The combination of intensity and extent was scored. Scores 2–3 were defined as a positive staining pattern, as shown in **Supplementary Table 2**. In addition, the score was also sub-classified into low (score 0-1) and high expression (score 2-3). Assessment of scoring was performed by two pathologists. If agreement was not obtained between the pathologists, we asked an additional pathologist regarding the assessment. Finally, the score was determined by agreement of more than two pathologists.

In the present study, a wide range of expression levels was observed for all the markers. Thus, we selected the deepest invasive region as a target area to measure the expression levels of markers.

### Hierarchical Analysis of the Expression of CAF and EMT Markers

Hierarchical cluster analysis was performed for clustering of the samples according to the expression level in order to achieve maximal homogeneity for each group and the greatest differences between the groups using open-access clustering software (Cluster 3.0 software; bonsai.hgc.jp/~mdehoon/software/cluster/software.htm). The clustering algorithm was set to centroid linkage clustering, which is the standard hierarchical clustering method used in biological studies.

### Statistical Analysis

Data were analyzed using JMP Pro 13.0 software (SAS, Tokyo, Japan). Data obtained for clinicopathological features (sex, location, pT, stage, histological type, lymphatic invasion, venous invasion, tumor budding, desmoplastic reaction, overall survival, disease-free survival) and subgroup (subgroups 1, 2 and 3) were analyzed using Fisher’s exact test. In addition, the comparison of the age distributions within each subgroup was performed using the Kruskal-Wallis test. If multigroup comparisons were needed for statistical analysis, we used Bonferroni corrections.

Kaplan-Meier analyses were performed using a log-rank test for survival analyses. Univariate and multivariate analyses were conducted with Cox proportional hazards model to identify statistical differences for prediction of overall and disease-free survival. The level of significance was *p <* 0.05, and the confidence interval (CI) was determined at the 95% level.

## Results

**Figure 1:**
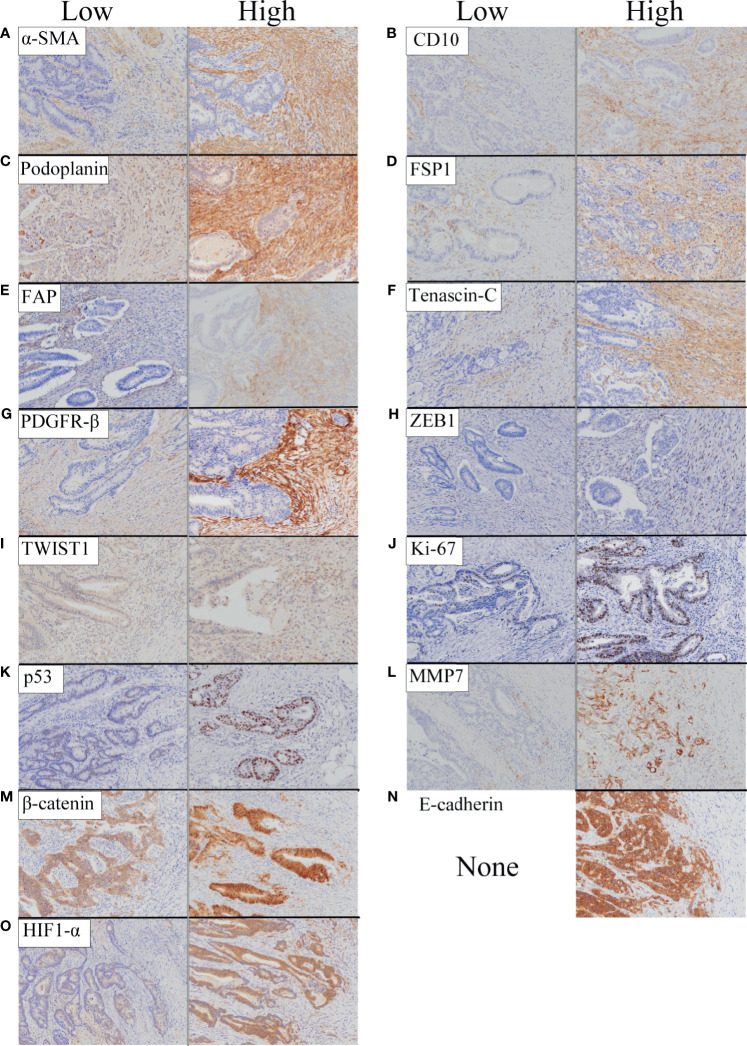
Representative features of immunohistochemical staining of biological markers we examined based on expression level (low and high). **(A)** α-SMA. **(B)** CD10. **(C)** Podoplanin. **(D)** FSP1. **(E)** FAP. **(F)** Tenascin-C. **(G)** PDGFR-β. **(H)** ZEB1. **(I)** TWIST1. **(J)** Ki-67. **(K)** p53. **(L)** MMP7. **(M)** β-catenin. **(N)** E-cadherin. **(O)** HIF1-α.

A representative figure is shown in **Figure 1**. In addition, the cancer invasive front is depicted in **Supplementary Figure 1**.

## Analyses of Clinicopathological Variables and Biological Markers in the First Cohort

### Hierarchical Clustering Based on Marker Scores in First Cohort

**Figure 2:**
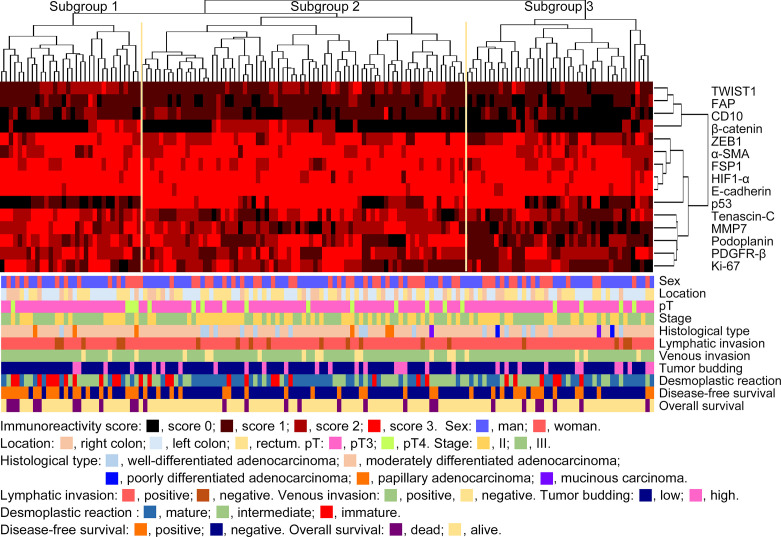
Hierarchical cluster analysis of colorectal cancer patients with stage II or III disease based on the expression patterns of cancer cells and cancer-associated fibroblast (CAF) proteins in the first cohort. The examined CRCs were subclassified into 3 subgroups.

We performed hierarchical clustering based on marker scores to evaluate differences in expression patterns of cancer cell-, CAF- and EMT-related markers in stage II and III CRC. Three distinct subgroups were stratified, as shown in **Figure 2**. The vertical line shows the expression of each marker in cancer cells and fibroblasts and the horizontal lines denote “relatedness” between samples. There was no statistical difference in the frequency of clinicopathological variables among subgroups 1, 2 and 3. Although immature desmoplastic reaction present in subgroup 1 showed a high frequency among the 3 subgroups, such association between the 3 subgroups did not quite reach a statistically significant level (*p* = 0.0508). However, the frequency of disease-free survival was significantly higher in subgroup 1 than in subgroup 2 (*p*<0.0001). Detailed data are shown in **Table 2**.

**Table 2:** Clinicopathological variables according to each subgroup in the first cohort.

		Subgroup 1 (%)		Subgroup 2 (%)		Subgroup 3 (%)		*p* value
Total		32		74		42		
Age median (range) (y)		69.0 (43–94)		67.0 (34–92)		67.5 (42–88)		0.1971
Sex	Man	19 (59.4)		45 (60.8)		26 (61.9)		1.0000
	Woman	13 (41.6)		29 (39.2)		16 (38.1)		
Location	Right colon	7 (21.9)		15 (20.3)		8 (19.0)		0.9516
	Left colon	15 (46.9)		30 (40.5)		19 (45.2)		
	Rectum	10 (31.2)		29 (39.2)		15 (35.7)		
pT	pT3	28 (87.5)		63 (85.1)		38 (90.5)		0.7678
	pT4	4 (12.5)		11 (14.9)		4 (9.5)		
Stage	II	13 (40.6)		37 (50.0)		21 (50.0)		0.6456
	III	19 (59.4)		37 (50.0)		21 (50.0)		
Histological type	WDA	1 (3.1)		10 (13.5)		6 (14.3)		0.1125
	MDA	28 (87.5)		60 (81.1)		33 (78.6)		
	PDA	0 (0.0)		0 (0.0)		2 (4.8)		
	PAP	3 (9.4)		3 (4.1)		0 (0.0)		
	MUC	0 (0.0)		1 (1.4)		1 (2.4)		
Lymphatic invasion	Positive	27 (84.4)		65 (87.8)		38 (90.5)		0.7149
	Negative	5 (15.6)		9 (12.2)		4 (9.5)		
Venous invasion	Positive	29 (90.6)		61 (82.4)		39 (92.9)		0.2694
	Negative	3 (9.4)		13 (17.6)		3 (7.1)		
Tumor budding	Low	28 (87.5)		58 (78.4)		31 (73.8)		0.3738
	High	4 (12.5)		16 (21.6)		11 (26.2)		
Desmoplastic reaction	Mature	8 (25.0)		36 (48.6)		21 (50.0)		0.0508
	Intermediate	11 (34.4)		27 (36.5)		15 (35.7)		
	Immature	13 (40.6)		11 (14.9)		6 (14.3)		
Disease-free survival	Positive	20 (62.5)*		15 (20.3)*		15 (35.7)		0.0002
	Negative	12 (37.5)		59 (79.7)		27 (64.3)		
Overall survival	Dead	10 (31.6)		10 (13.5)		7 (16.7)		0.0999
	Alive	20 (62.5)		64 (86.5)		35 (83.3)		

WDA, well-differentiated adenocarcinoma; MDA, moderately differentiated adenocarcinoma; PDA, poorly differenced adenocarcinoma; PAP, papillary carcinoma; MUC, mucinous carcinoma; *p < 0.0001.

### Survival Analyses of Each Subgroup in the First Cohort

Kaplan–Meier analyses were performed to determine the association between the disease-free survival frequencies and the subgroups. Subgroup 1 had a poorer disease-free survival, compared to subgroup 2 (p < 0.0001). However, overall survival did not differ among the subgroups (**Supplementary Figure 2**).

### The Association of Clinicopathological Variables and Subgroups With Survival of Stage II and III CRC Patients: Univariate and Multivariate Analyses of the First Cohort Using a Cox Proportional Hazards Model

The univariate analysis of stage II and III CRC patients (**Table 3a**) identified 5 factors: histologic type (mucinous carcinoma vs. well differentiated adenocarcinoma), stage (II *vs* III), desmoplastic reaction (mature *vs* immature) and subgroup (1 *vs* 2; 1 *vs* 3). **Table 3b** reveals that 3 factors (mucinous carcinoma *vs* well differentiated adenocarcinoma, mature DR *versus* immature DR, subgroup 1 *versus* 2) were retained in the multivariate analysis using a Cox proportional hazards model

**Table 3:** Association of clinicopathological variables and subgroups with disease-free survival and overall survival in the first cohort in univariate and multivariate analyses.

Variables		a. Univariate analysis			b. Multivariate analysis			c. Univariate analysis			d. Multivariate analysis		
		HR	95%CI	*p* value	HR	95%CI	*p* value	HR	95%CI	*p* value	HR	95%CI	*p* value
		Disease-free survival						Overall survival					
Sex	Woman *vs* man	1.420	0.807-2.477	0.2207				1.456	0.674-3.126	0.3335			
Location	Rectum *vs* Left colon	1.488	0.784-2.864	0.2238				1.336	0.549-3.429	0.5257			
	Right colon *vs* Left colon	1.671	0.778-3.476	0.1821				1.411	0.492-3.950	0.5104			
	Right colon *vs* Rectum	1.123	0.534-2.260	0.7508				1.056	0.393-2.627	0.9084			
pT	pT4 *vs* pT3	1.488	0.646-3.007	0.3261				2.477	0.967-5.630	0.0581			
Stage	III vs II	2.319	1.299-4.322	0.0041	1.816	0.981-3.520	0.0577	2.575	1.139-6.570	0.0222	2.264	0.972-5.923	0.0586
Histological type	MUC *vs* WDA	10.443	1.857-58.726	0.0253	9.556	1.537-59.396	0.0350	4.994	0.448-55.676	0.2455			
Lymphatic invasion	Positive *vs* Negative	2.444	0.895-10.069	0.0865				3.596	0.762-64.238	0.1226			
Venous invasion	Positive *vs* Negative	1.473	0.643-4.255	0.3878				1.677	0.499-10.431	0.4493			
Tumor budding	High *vs* Low	1.386	0.707-2.548	0.3275				1.502	0.636-3.299	0.3383			
Desmoplastic reaction	Immature *vs* Intermediate	1.766	0.894-3.446	0.1003				1.788	0.725-4.257	0.2004			
	Immature *vs* Mature	3.023	1.486-6.183	0.0025	2.190	1.012-4.793	0.0467	3.660	1.316-10.947	0.0132	2.473	0.821-7.870	0.107
	Intermediate *vs* Mature	1.711	0.869-3.435	0.1200				2.048	0.793-5.893	0.1407			
Subgroup	Subgroup 1 *vs* 2	3.626	1.860-7.224	0.0002	3.012	1.512-6.127	0.0018	2.643	1.082-6.459	0.0335	2.082	0.825-5.261	0.1185
	Subgroup 1 *vs* 3	2.024	1.036-4.049	0.0392	1.710	0.819-3.672	0.1538	2.058	0.789-5.676	0.1393			
	Subgroup 3 *vs* 2	1.791	0.868-3.697	0.1135				1.284	0.466-3.347	0.6154			

WDA, well-differentiated adenocarcinoma; MUC, mucinous carcinoma; HR, hazard ratio; 95%CI, 95% confidence interval.

Using a similar method, we performed univariate analysis for screening of overall survival of stage II and III CRC patients. As a result, 3 factors, including stage (II *vs* III), desmoplastic reaction (mature *vs* immature), and subgroup (1 *vs* 2) were identified in univariate analysis (**Table 3c**). However, no factors were retained in multivariate analysis (**Table 3d**).

### Association of Individual Markers With Individual Subgroups in the First Cohort

**Figure 3:**
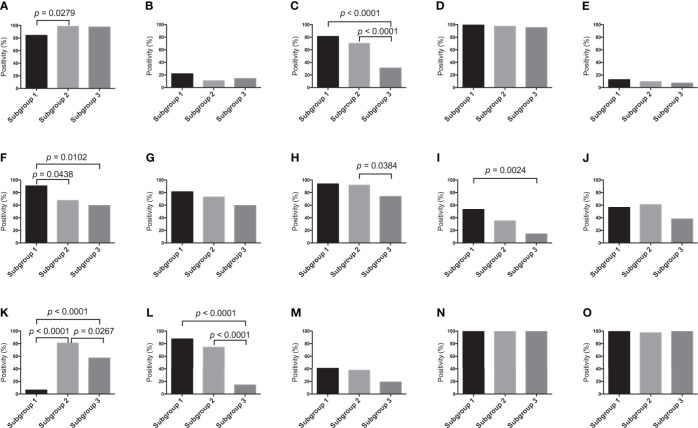
Marker expression levels. **(A)** Expression level of each marker in the first cohort. **(A)** α-SMA, **(B)** CD10, **(C)** Podoplanin, **(D)** FSP1, **(E)** FAP, **(F)** Tenascin- C, **(G)** PDGFR-β, **(H)** ZEB1, **(I)** TWIST, **(J)** Ki-67, **(K)** p53, **(L)** MMP7, **(M)** β-catenin, **(N)** E-cadherin and **(O)** HIF1-α.

The frequency of positive scores (score 2 or 3) of SMA was higher in subgroup 2 than in subgroup 1. There were statistically significant differences in the frequencies of positive scores among subgroups 1, 2 and 3 (subgroup 1, 2 > 3). In addition, significant differences in the frequencies of positive scores for tenascin-C between subgroups 1 and 2, and 3 were found (subgroup 1 > 2, 3). The frequency of the positive score for ZEB1 was statistically higher in subgroup 2 than in subgroup 3. Next, there was a statistically significant difference in the frequencies of positive scores for TWIST1 between subgroup 3 and subgroup 1 (subgroup 1 > 3). The positive score for p53 was significantly greater in subgroup 2 than in subgroups 1 and 3. Furthermore, there was a significant difference in the frequencies of positive scores for p53 between subgroups 1 and 3. Finally, we observed statistically significant differences in the frequencies of positive MMP7 scores among subgroups 1 and 2, and 3 (subgroup 1, 2 > 3). Detailed data are shown in **Figure 3**.

### The Association of Clinicopathological Variables and Individual Markers With the Survival of Stage II and III CRC Patients: Univariate and Multivariate Analyses of the First Cohort

With regard to disease-free survival, 3 variables (stage II *vs* III; mature *vs* immature; mucinous carcinoma *vs* well differentiated adenocarcinoma) and one marker (tenascin-C) were identified in univariate analysis (**Table 4a**). Among those 4 parameters, 2 variables, including desmoplastic reaction and histological type and one marker, tenascin-C, were retained in multivariate analysis (**Table 4b**). In overall survival, stages (II *vs* III) and desmoplastic reaction (mature *vs* immature) were identified in univariate analysis (**Table 4c**). Desmoplastic reaction (mature *vs* immature) was retained in multivariate analysis (**Table 4d**).

**Table 4:** Association of clinicopathological variables and individual marker with disease-free survival and overall survival in the first cohort in univariate and multivariate analyses.

Variables		a. Univariate analysis			b. Multivariate analysis			c. Univariate analysis			d. Multivariate analysis		
		HR	95%CI	*p* value	HR	95%CI	*p* value	HR	95%CI	*p* value	HR	95%CI	*p* value
		Disease-free survival						Overall survival					
Stage	III *vs* II	2.319	1.299-4.322	0.0041	1.760	0.943-3.430	0.0762	2.575	1.139-6.570	0.0222	2.237	0.959-5.856	0.0629
Histological type	MUC *vs* WDA	10.443	1.857-58.726	0.0253	15.097	2.505-90.973	0.0132	5.001	0.929-33.667	0.2451			
Desmoplastic reaction	Immature *vs* Intermediate	1.766	0.894-3.446	0.1003				1.788	0.725-4.257	0.2004			
	Immature *vs* Mature	3.023	1.486-6.183	0.0025	2.358	1.115-5.033	0.0250	3.660	1.316-10.947	0.0132	3.076	1.085-9.352	0.0347
	Intermediate *vs* Mature	1.711	0.869-3.435	0.1200				2.048	0.793-5.893	0.1407			
α-SMA	Positive *vs* Negative	0.684	0.250-2.821	0.5468				0.628	0.187-3.905	0.5532			
CD10	Positive *vs* Negative	1.690	0.798-3.254	0.1606				1.412	0.472-3.453	0.5025			
Podoplanin	Positive *vs* Negative	1.019	0.583-1.821	0.9477				1.039	0.486-2.307	0.9215			
FSP1	Positive *vs* Negative	0.677	0.209-4.150	0.6111				0.357	0.072-6.461	0.3876			
FAP	Positive *vs* Negative	0.501	0.120-1.392	0.2067				0.278	0.154-1.334	0.1270			
Tenascin-C	Positive *vs* Negative	2.694	1.293-6.557	0.0065	2.317	1.068-5.870	0.0324	2.052	0.840-6.130	0.1201			
PDGFR-β	Positive *vs* Negative	0.988	0.550-1.863	0.9680				0.764	0.351-1.786	0.5176			
ZEB1	Positive *vs* Negative	1.269	0.584-3.325	0.5730				2.094	0.623-13.018	0.2638			
TWIST1	Positive *vs* Negative	1.512	0,851-2.639	0.1554				1.185	0.533-2.540	0.6673			
Ki-67	Positive *vs* Negative	1.139	0.653-2.011	0.6477				1.245	0.579-2.736	0.5754			
p53	Positive *vs* Negative	0.629	0.359-1.098	0.1023				0.504	0.231-1.077	0.0767			
MMP7	Positive *vs* Negative	1.621	0.890-3.069	0.1096				1.205	0.560-2.734	0.6383			
β-catenin	Positive *vs* Negative	0.770	0.401-1.395	0.3974				0.979	0.419-2.127	0.9578			
E-cadherin*													
HIF1-α	Positive *vs* Negative	0.461	0.101-8.172	0.4961				0.297	0.062-5.344	0.3187			

WDA, Well-differentiated adenocarcinoma; MUC, mucinous carcinoma; HR, Hazard ratio; 95%CI, 95%confidence interval. *could not analyze, why all cases were positive expression of the marker.

## Analyses of Clinicopathological Variables and Individual Markers in the Second Cohort (Validation)

### The Association of Clinicopathological Variables and Individual Markers With the Survival of Stage II and III CRC Patients: Univariate and Multivariate Analyses of the Second Cohort

With regard to disease-free survival, 5 variables (pT3 *vs*. pT4; stage II *vs*. III; positive venous invasion *vs*. negative venous invasion; low grade budding *vs*. high grade budding; mature *vs*. immature) and 2 markers (tenascin-C and β-catenin) were identified in univariate analysis (**Table 5a**). However, only 1 factor (tenascin-C) was retained in multivariate analysis (**Table 5b**). In overall survival, 4 variables (pT3 *vs*. pT4; stage II vs. III and desmoplastic reactions (mature *vs*. immature; and intermediate *vs*. mature) and 2 individual markers (tenascin-C and Ki-67) were detected in univariate analysis (**Table 5c**). Only the positive expression of tenascin-C was retained in multivariate analysis (**Table 5d**).

**Table 5:** Association of clinicopathological variables and individual markers with disease-free survival and overall survival in the second cohort in univariate and multivariate analyses.

Variables		a. Univariate analysis			b. Multivariate analysis			c. Univariate analysis			d. Multivariate analysis		
		HR	95%CI	*p* value	HR	95%CI	*p* value	HR	95%CI	*p* value	HR	95%CI	*p* value
		Disease-free survival						Overall survival					
Sex	Woman *vs* Man	0.819	0.430-1.512	0.5283				0.919	0.395-2.048	0.8320			
Location	Rectum *vs* Left colon	1.982	0.976-4.345	0.0588				1.617	0.639-4.603	0.3180			
	Right colon *vs* Left colon	1.094	0.417-2.774	0.8505				1.088	0.313-3.613	0.8894			
	Right colon *vs* Rectum	1.813	0.850-4.310	0.1278				1.486	0.560-4.632	0.4393			
pT	pT4 *vs* pT3	2.279	1.142-4.307	0.0208	1.527	0.714-3.112	0.2662	2.630	1.065-5.986	0.0369	1.936	0.746-4.670	0.1667
Stage	III *vs* II	3.114	1.633-6.334	0.0004	1.991	0.977-4.301	0.0581	4.599	1.847-13.885	0.0007	2.619	0.980-8.321	0.0552
Histological type	WDA *vs* MDA	0.627	0.264-1.492	0.2657				0.787	0.269-2.303	0.6533			
Lymphatic invasion	Positive *vs* Negative	4.334	0.944-76.833	0.0620				2.447	0.516-43.777	0.3125			
Venous invasion	Positive *vs* Negative	4.855	1.057-86.076	0.0401	4.073	0.838-73.423	0.0904	2.722	0.574-48.690	0.2501			
Tumor budding	High *vs* Low	2.291	1.170-4.285	0.0169	1.667	0.825-3.237	0.1497	2.090	0.847-4.752	0.1052			
Desmoplastic reaction	Immature *vs* Intermediate	1.520	0.712-3.204	0.2736				1.227	0.451-3.118	0.6754			
	Immature *vs* Mature	2.322	1.076-4.976	0.0324	0.993	0.415-2.340	0.9867	3.293	1.092-10.245	0.0348	1.596	0.493-5.305	0.4312
	Intermediate *vs* Mature	1.527	0.733-3.202	0.2558				2.684	1.021-7.793	0.0452	1.895	0.696-5.686	0.2143
α-SMA	Positive *vs* Negative	1.423	0.437-8.736	0.6078				1.580	0.333-28.264	0.6303			
CD10	Positive *vs* Negative	0.657	0.267-1.391	0.2883				0.704	0.295-1.941	0.4698			
Podoplamin	Positive *vs* Negative	0.752	0.399-1.496	0.4020				0.564	0.251-1.343	0.1872			
FSP1	Positive *vs* Negative	0.355	0.108-2.185	0.2181				0.296	0.061-5.139	0.3172			
FAP	Positive *vs* Negative	0.819	0.425-1.520	0.5323				4.045	0.853-72.358	0.0866			
Tenascin-C	Positive *vs* Negative	5.025	2.164-14.621	0.0001	3.973	1.656-11.795	0.0012	4.527	1.559-19.172	0.0036	3.188	1.038-13.882	0.0421
PDGFR-β	Positive *vs* Negative	1.290	0.685-2.566	0.4395				1.416	0.611-3.661	0.4288			
ZEB1	Positive *vs* Negative	0.683	0.248-2.825	0.5463				0.562	0.166-3.510	0.4713			
TWIST1	Positive *vs* Negative	1.067	0.581-1.971	0.8331				1.237	0.553-2.818	0.6028			
Ki-67	Positive *vs* Negative	1.667	0.885-3.316	0.1157				2.952	1.187-8.906	0.0185	2.114	0.834-6.465	0.1191
p53	Positive *vs* Negative	1.362	0.730-2.663	0.3378				1.255	0.552-3.096	0.5955			
MMP7	Positive *vs* Negative	0.651	0.354-1.199	0.1671				0.823	0.368-1.875	0.6358			
β-catenin	Positive *vs* Negative	2.119	1.143-4.084	0.0169	1.502	0.796-2.940	0.2121	1.865	0.830-4.440	0.1323			
E-cadherin*													
HIF1-α*													

WDA, Well-differentiated adenocarcinoma; MUC, mucinous carcinoma; HR, Hazard ratio; 95%CI, 95%confidence interval. *Could not analyze, why all cases were positive expression of the marker.

## Discussion

Certain proteins expressed by microenvironmental cells play crucial roles in neoplastic progression of CRC. Those proteins may be derived from cancer cells or from stromal cells (sometimes termed “cancer-associated fibroblasts” (CAFs) (12, 13). Proteins expressed by cancer cells and CAFs interact with one another and this interaction is likely important at the invasive front (12, 13). According to that theory, the combination of proteins from cancer cells and CAFs mediate tumor growth and progression (12, 13). In the present study, specific expression patterns could be correlated with the prognosis of stage II and III CRC patients. Therefore, the current results suggest that a specific subgroup (identified here by stratification) can be used to evaluate the role and significance of various proteins produced by microenvironmental cells. Finally, in the present study, subgroup 1 was correlated with disease-free survival. However, the presence in subgroup 1 did not correlate with overall survival. The reason remains unknown.

In the current study, we used 15 microenvironment-related markers (cancer cell markers and CAF markers) to identify associations of expression patterns with patient outcomes. Among the cancer cell-related markers, a high Ki-67-positive rate and overexpression of p53 were considered to reflect the characteristics of tumors. Intranuclear expression of β-catenin and high expression of MMP7, E-cadherin, and HIF1-α are closely associated with tumor budding, which is a key histological feature occurring in the cancer microenvironment (16–18). By contrast, stromal markers, including α-SMA, CD10, podoplanin, FSP1, PDGFR β, FAP, and TNC, were used as CAF markers. These markers are thought to be associated with enhanced progression of CAFs. Based on these findings, we suggest that the microenvironment-related markers used in the current study may be suitable for identification of the molecular mechanisms of neoplastic progression and cancer metastasis in the tumor microenvironment.

Tenascin-C (TNC) is an extracellular matrix molecule that drives the progression of many types of human cancer. The basis for its actions remains unclear (19). TNC is associated with organogenesis accompanying cell proliferation and migration, resulting in the epithelial-mesenchymal transition (EMT) that might result from interactions between cancer cells and stromal cells (20). EMT is the process by which polarized epithelial cells are converted into mesenchymal cells during cancer progression. As a result, carcinoma cells lose their epithelial polarity and intercellular connections, allowing them to escape the surrounding epithelium (20, 21). The expression of TNC facilitates such phenotypic changes, alterations that are enhanced by TGF-β, a promoter of EMT (19–21). Murakami et al. revealed that TNC in primary CRC stroma might be a novel biomarker that is predictive of postoperative prognosis (21). Finally, TNC may promote EMT-like change and proliferation, alterations that lead to poor prognosis in CRC patients (20).

TNC may be involved in cancer growth and metastatic processes *via* the Hedgehog (HH) signaling pathway, caused either by mutations in the pathway (ligand independent) or through HH overexpression (ligand dependent) (22). HH signaling starts with secretion of the HH ligand, followed by secretion of Patched (PTC), the transmembrane protein Smoothened (SMO) and three GLI (Glioma-associated oncogene) zinc finger transcription factors (23). The HH/GLI1 pathway promotes cancer growth, stem cell self-renewal and metastatic behavior in advanced CRC (24). Human CRC stem cells require active HH/GLI1 signaling for survival and self-renewal (25). Our finding suggests that activation of CAF at the invasive front is caused by high expression of TNC facilitated *via* HH signaling (26). In addition, accumulating evidence suggests that activated HH signaling plays an important role in neoplastic transformation as well as the development of drug resistance of human cancers (27). Thus, HH signaling during tumorigenesis and the development of chemo-resistance are closely associated. Those findings suggest that therapeutic strategies might target such signals in human cancers and their relapse (26, 27). For example, cyclopamine is an HH signal pathway antagonist and consequently is expected to improve the survival of patients with CRC by inhibiting the proliferation of colon cancer cells (28). Previous study showed that cyclopamine treatment results in decreased levels of mRNA coding for HH, SMO and PTCH, all of which were highly expressed in colon cancer cell lines (28). These findings may influence potential therapeutic strategies because TNC expression by CAF may be targeted in future molecular therapies.

High expression of TNC was reported to be a prognostic marker for CRC through induction of EMT and cell proliferative activity (20). According to that study, TNC may facilitate EMT-like changes and could be associated with a poor prognosis of CRC patients. This finding is consistent with other data showing that cancer cell-derived TNC promotes cancer cell invasion *via* EMT regulation. Thus, it is a novel indicator of poor prognosis (29). In the present study, we found that even in stages II and III, intermediate stages that account for the majority of surgically resected CRC, TNC was an independent prognostic marker. This result was validated by analysis of a second cohort. The present results showed that TNC in primary CRC stroma has the potential to be a novel biomarker that predicts postoperative prognosis.

There are some limitations to this study. First, the immunohistochemical markers we used in the present study may not yield consistent results. For clinical application, immunohistochemical reagents must be reliable and reproducible. In that regard, many immunohistochemical markers that are closely associated with the formation of the microenvironment have been analyzed (12, 13). In the current study, 15 microenvironment-related markers, including Ki-67, p53, β-catenin, MMP7, E-cadherin, and HIF1-α (for cancer cells) and CD10, podoplanin, FSP 1, PDGFR β, FAP, TNC, ZEB1, and TWIST1 (for CAFs) were used. Briefly, Ki-67 positivity and p53 overexpression have been widely used as characteristics of tumors. The remaining factors, including β-catenin, MMP7, E-cadherin, and HIF1-α, are closely associated with the formation of the cancer microenvironment. In addition, stromal factors could be classified as CAF or EMT markers. The two stromal markers used in this study were considered CAF markers given that all markers we used were expressed in CAFs. These CAF markers are suitable for identifying the functions of CAFs. Therefore, we concluded that the immunohistochemical markers examined in this study were all involved in generation of the tumor microenvironment at the invasive front. Finally, analysis of these immunohistochemical markers should yield reliable and reproducible results, as demonstrated in the current study. Second, the heterogeneous expression of the markers examined in this study may be problematic when determining marker expression levels (30). Although it may be difficult to avoid this problem, we suggest that the invasive front of cancer cells, which is critical for tumor progression, may be the best region for measuring the immunohistochemical expression levels of the chosen markers (10, 11). Finally, although there are many different reports regarding prognostic factors in CRC (31, 32), the different results may reflect the choice of markers, patient stage, heterogeneity of expression, staining platform, judging methods and cut-off value. In the present study, we suggest that the current results are reliable and reproducible under the conditions we employed.

## Conclusions

Cancer cells and CAFs express many proteins that modulate neoplastic progression and metastasis. In the present study, we found that specific expression patterns may allow the prediction of patient outcome in CRC. In addition, the expression of TNC by CAFs might be a potential prognostic biomarker in stage II and III CRC patients. These results highlight a potential role for TNC in CRC tumor progression and provide novel mechanistic insights into the roles of HH, as it is associated with high expression of TNC in driving CRC progression. Our findings also suggest that TNC could be a critical target gene for the treatment of CRC. However, further study will be needed in the near future to confirm these results.

## Data Availability Statement

The raw data supporting the conclusions of this article will be made available by the authors, without undue reservation.

## Ethics Statement

The studies involving human participants were reviewed and approved by the ethics committee of Iwate Medical University Hospital (approval number MH2020-070). The patients/participants provided their written informed consent to participate in this study. Written informed consent was obtained from the individual(s) for the publication of any potentially identifiable images or data included in this article.

## Author Contributions

MH, who is the first author, constructed the figures and tables and performed the statistical analyses. MO assisted statistical analyses. YK and HU supported pathological interpretation of desmoplastic reactions. NY and NU helped in the interpretation of pathological findings. KO and AS provided clinical support during the preparation of the manuscript. TS, who is the corresponding author, contributed to the preparation of the manuscript, including all aspects of the data collection and analysis. All authors contributed to the article and approved the submitted version.

## Conflict of Interest

The authors declare that the research was conducted in the absence of any commercial or financial relationships that could be construed as a potential conflict of interest.

## Publisher’s Note

All claims expressed in this article are solely those of the authors and do not necessarily represent those of their affiliated organizations, or those of the publisher, the editors and the reviewers. Any product that may be evaluated in this article, or claim that may be made by its manufacturer, is not guaranteed or endorsed by the publisher.

## Supplementary Material

Supplementary Figure 1 Histological features of the invasive front. **(A)** Low-power view of the invasive area of CRC. **(B)** High-power view of the invasive area of CRC (mature type desmoplastic reaction). **(C)** Low-power view of the invasive area of CRC. **(D)** High-power view of the invasive area of CRC (intermediate type desmoplastic reaction). **(E)** Low-power view of the invasive area of CRC. **(F)** High-power view of the invasive area of CRC (immature type desmoplastic reaction).

Supplementary Figure 2 Kaplan-Meier analyses of the disease-free survival **(A)** and overall survival **(B)** based on each subgroup of the first cohort.

## Abbreviations

CAF, Cancer associated fibroblast; CRC, colorectal cancer; TMA, tissue microarray; MMP7, matrix metalloproteinase-7; FSP1, fibroblast specific protein 1; PDGFR-b, platelet derived growth factor receptor beta; FAP, fibroblast associated protein; ZEB1, zinc finger E-box binding homeobox 1; TWIST1, twistrelated protein 1.
