# Supplementary figures and images for: Expression Patterns of Microenvironmental Factors and Tenascin-C at the Invasive Front of Stage II and III Colorectal Cancer: Novel Tumor Prognostic Markers
Source: Front Oncol. 2021 Aug 19;11:690816. doi: 10.3389/fonc.2021.690816 (PMC8417423; doi:10.3389/fonc.2021.690816)

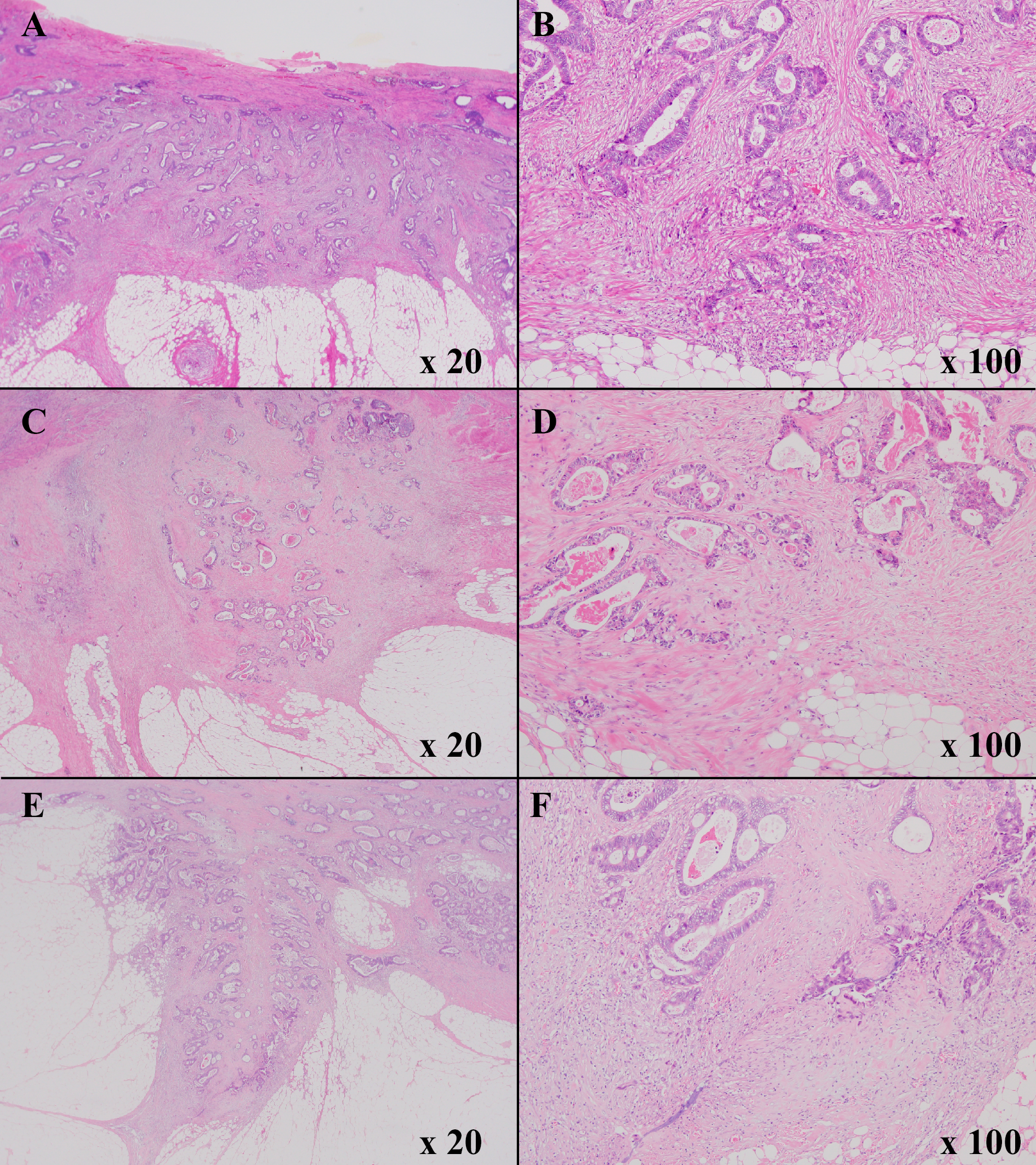

Supplement: Supplementary Figure 1 — Histological features of the invasive front. (A) Low-power view of the invasive area of CRC. (B) High-power view of the invasive area of CRC (mature type desmoplastic reaction). (C) Low-power view of the invasive area of CRC. (D) High-power view of the invasive area of CRC (intermediate type desmoplastic reaction). (E) Low-power view of the invasive area of CRC. (F) High-power view of the invasive area of CRC (immature type desmoplastic reaction). [file Image_1.tif]

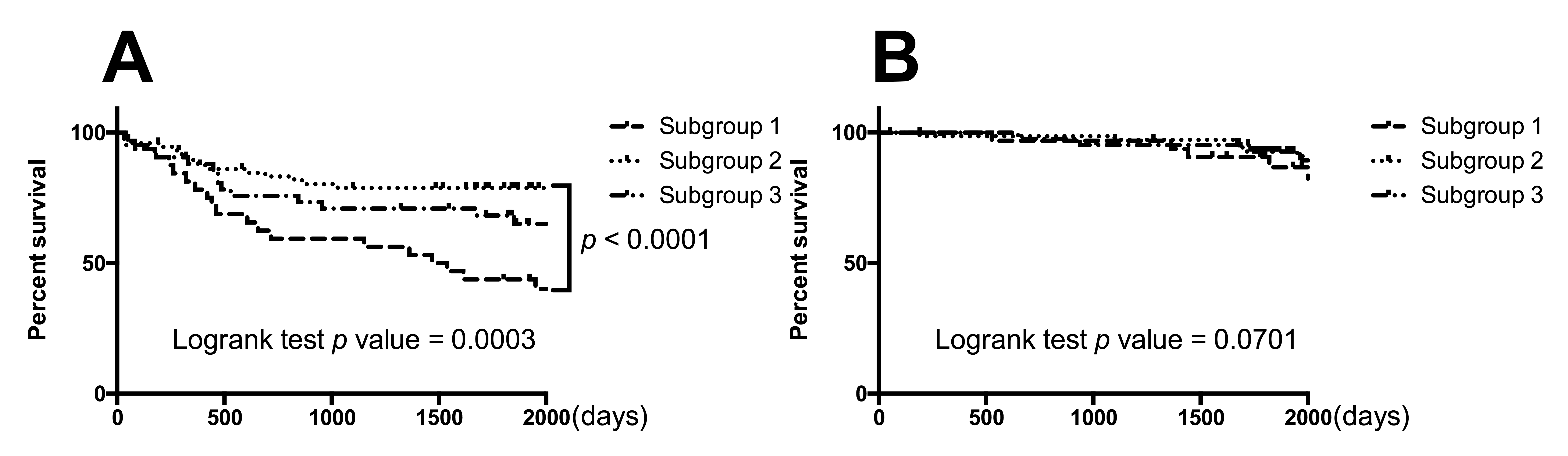

Supplement: Supplementary Figure 2 — Kaplan-Meier analyses of the disease-free survival (A) and overall survival (B) based on each subgroup of the first cohort. [file Image_2.tiff]
