# Supplementary material for: Expression Patterns of Microenvironmental Factors and Tenascin-C at the Invasive Front of Stage II and III Colorectal Cancer: Novel Tumor Prognostic Markers
Source: Front Oncol. 2021 Aug 19;11:690816. doi: 10.3389/fonc.2021.690816 (PMC8417423; doi:10.3389/fonc.2021.690816)
Supplement: Supplementary file 3 [file Table_1.docx]

Supplementary Table 1. Antibodies used in the present study.

| Antibody | Source | Clone | Dilution | Treatment |
| --- | --- | --- | --- | --- |
| α-SMA | Dako | 1A4 | Ready to use | Heat retrieval (pH9.0) |
| CD10 | Dako | 56C6 | Ready to use | Heat retrieval (pH9.0) |
| Podoplanin | Dako | D2-40 | Ready to use | Heat retrieval (pH9.0) |
| FSP1 | Dako | Polyclonal | 1:400 | Heat retrieval (pH6.0) |
| FAP | Abcam | EPR20021 | 1:250 | Heat retrieval (pH9.0) |
| Tenascin-C | IBL | 4F10TT | 1:200 | Heat retrieval (pH6.0) |
| PDGFR-β | CST | 28E1 | 1:100 | Heat retrieval (pH9.0) |
| ZEB1 | Sigma-Aldrich | Polyclonal | 1:200 | Heat retrieval (pH6.0) |
| TWIST1 | Abcam | Twist2C1a | 1:500 | Heat retrieval (pH9.0) |
| Ki-67 | Dako | MIB1 | Ready to use | Heat retrieval (pH9.0) |
| p53 | Dako | DO7 | Ready to use | Heat retrieval (pH9.0) |
| MMP7 | Daiichi fine chemical | 141-7B2 | 1:100 | Heat retrieval (pH9.0) |
| β-catenin | Dako | β-Catenin-1 | Ready to use | Heat retrieval (pH9.0) |
| E-cadherin | Dako | NCH-38 | Ready to use | Heat retrieval (pH9.0) |
| HIF1-α | Novus Biologicals | Polyclonal | 1:100 | Heat retrieval (pH6.0) |

Supplementary Table 2. Immunohistochemical scoring according to immunostaining intensity and area.

| Staining  intensity | Staining area (%) | |  | |  | |
| --- | --- | --- | --- | --- | --- | --- |
|  | 0 | 0 < area ≤ 25 | | 25 < area ≤ 50 | | 50 < area ≤ 100 |
| Negative | Score 0 | Score 0 | | Score 0 | | Score 0 |
| Weak | Score 0 | Score 1 | | Score 1 | | Score 2 |
| Moderate | Score 0 | Score 1 | | Score 2 | | Score 3 |
| Strong | Score 0 | Score 2 | | Score 3 | | Score 3 |
